# Unattributed Medicare beneficiaries have greater social disadvantage than those linked to primary care physicians

**DOI:** 10.1093/haschl/qxag198

**Published:** 2026-08-01

**Authors:** Katherine M Ianni, Laura A Hatfield

**Affiliations:** Division of Health Policy and Economics, Department of Population Health Sciences, Weill Cornell Medical College, New York, NY 10022, United States; Statistics & Data Science Department, NORC at the University of Chicago, Chicago, IL, United States

**Keywords:** health equity, alternative payment models, payment reform, Medicare

## Abstract

**Introduction:**

Attributing patients to physicians using claims data is a key design feature of most payment and delivery models. However, claims-based attribution makes model participants less representative by excluding beneficiaries who use less care.

**Methods:**

We used the attribution methodology from the largest completed primary care model, Comprehensive Primary Care Plus (CPC+), to describe differences in attributable and unattributable Medicare beneficiaries living in CPC + regions from 2016 to 2021.

**Results:**

Unattributed beneficiaries were more male (56% vs 43%), eligible for the low-income drug subsidy (15% vs 12%), dual eligible (13% vs 10%), and non-White (22% vs 15%).

**Conclusion:**

Tests of models that use claims-based attribution may fail to generate evidence relevant to underserved groups. Policymakers should consider attribution designs that could improve representativeness of model test populations.

## Introduction

The Centers for Medicare and Medicaid Innovation (CMMI) leads health care system transformation through the design and testing of innovative payment and delivery models. Many of the alternative payment models tested by CMMI aim to improve primary care delivery and financing. The focus on transforming primary care reflects the central role that primary care providers (PCPs) play in coordinating care and containing costs.^1^ One key feature of these models is the attribution of patients to providers.

The details of attribution are important for efforts to advance health equity in payment and delivery reform. Attribution determines who is exposed to model tests and, importantly, whose experiences are included in evaluations.^2^ Underserved groups who use less care^3-9^ are less likely to be attributed to model providers; therefore, utilization-based attribution can cause medically underserved groups to be disproportionately “unattributable.”^10,11^

Prior evidence shows variation in patients attributed across methods.^12^ One study of concordance between patient-identified PCPs in electronic health records and claims-based PCP attribution found misclassification ranging from 14% to 75%, with higher rates among non-White and dual-eligible people.^13^ In the Medicare Shared Savings Program, unattributable patients were more likely to be from minoritized racial and ethnic groups, live in high-poverty areas, and be near the end of life.^14^

The objective of this analysis is to assess how claims-based attribution impacts representativeness for the purposes of generating evidence to advance health equity in payment and delivery reform. We demonstrate this empirically by describing beneficiaries who are attributed and unattributable according to the CPC + model design. Using the CPC + attribution rules, we attribute beneficiaries to physicians and compare the demographics, health, and care utilization of attributed vs unattributed beneficiaries. We stratify by attribution method to highlight how changes to the algorithm could change the attributed population.

## Study data and methods

Our study period was 2016 to 2021, and we used data starting in 2014 to enable the required look-back period for attribution. We used the Master Beneficiary Summary File (MBSF) Base, Chronic Condition, and Cost and Use segments to measure beneficiary characteristics, clinical conditions, and utilization. We used the Medicare fee-for-service (FFS) outpatient and carrier claims files for attribution.

To construct a sample of beneficiaries eligible for attribution, we followed the beneficiary eligibility criteria used by the CPC+ model evaluators.^15^ We included Medicare FFS beneficiaries living in areas where CPC+ was offered and excluded beneficiaries who were not enrolled in Medicare Parts A and B for all 12 months of the year, were enrolled in Medicare Advantage at any point during the year, or died during the year.

To assign beneficiaries to providers, we followed the CPC+ attribution logic^15^ with 3 changes. First, we attributed beneficiaries to providers (NPIs) annually rather than quarterly for simplicity. Second, we attributed beneficiaries to NPIs rather than practice groups so we could use the same method for physicians not in CPC+. Third, we did not include voluntary alignment because this cannot be observed for non-CPC+ providers and beneficiaries. The model evaluators also excluded voluntarily aligned beneficiaries from the evaluation analyses for this reason and noted that fewer than half a percent of beneficiaries in the model were attributed based on voluntary alignment.^16^

Attribution required at least one eligible primary care service claim in the previous 24 months (eg, to attribute a beneficiary in 2016 required an eligible primary care claim in 2014-2015). Primary care service codes included those for office/outpatient visit evaluation and management (99 201-99 205 and 99 211–99 215), home care (99 324-99 328; 99 334–99 337; 99 339–99 345; and 99 347–99 350), Welcome to Medicare and Annual Wellness visits (G0402, G0438, G0439), advance care planning (99 497), collaborative care model (G0502–G0504 and 99 492, 99 493, 99 494), cognition and functional assessment for patient with cognitive impairment (G0505 and 99 483), outpatient clinic visit for assessment and management (G0463), and transitional care management services (99 495–99 496). The chronic condition management (CCM) services included CCM services (99 490 and 99 491), complex CCM services (99 487 and 99 488), assessment/care planning for patients requiring CCM services (G0506), Care management services for behavioral health conditions (G0507 and 99 484), and Prolonged services without face-to-face contact (99 358).^15^ In 2016-2017, we assigned beneficiaries to the NPI who billed their most recent CCM visit or, if there was no CCM visit, to the NPI who billed the plurality of their primary care services, as defined by the model methodology.^15^ In 2018, CPC+ added annual wellness visits (AWVs) as the primary mode of attribution. Therefore in 2018-2021, we assigned beneficiaries to the NPI who billed the most recent AWV or, if there was no AWV, as above. Finally, for beneficiaries attributed using a plurality of services, ties between NPIs were broken using the most recent claim or, if still tied, randomly.

Using the MBSF base, we recorded beneficiary characteristics, including the RTI race and ethnicity variable, dual eligibility for Medicare and Medicaid, eligibility for the low-income subsidy (LIS), eligibility for Medicare via disability, age, and sex. We measured health status using chronic condition flags from the MBSF Chronic Conditions files. From the MBSF Cost and Use file, we measured counts of emergency visits (inpatient and outpatient), total inpatient days and stays, Evaluation and Management (E&M) visits, and home health visits.

For beneficiaries in CPC+ regions who were eligible for the CPC+ model, we attributed to NPIs annually then pooled across 2016-2021 for our analytic sample. We report the number of benes unattributed, attributed, and by attribution method (AWV, CCM, or plurality).

Second, we summarized beneficiary characteristics and health status by attribution status and method (among attributed beneficiaries). Characteristics included sex, age, LIS and dual eligibility, disability, race and ethnicity, and chronic condition flags and are presented as means (for age) or percentages (the remaining variables).

Finally, we assessed the health care use patterns of unattributed and attributed beneficiaries. We compared the distribution of emergency care visits, inpatient days and stays, E&M visits, and home health visits. We summarized the distributions using quantiles and means.

### Limitations

We acknowledge the limitations of this study. We followed a modified version of the CPC+ attribution methodology used by the contracted model evaluators, and there could be small differences in our study sample compared to the exact evaluation sample because of this. However, given that the main objective of our analysis was to understand the representativeness of groups using claims-based attribution, and we applied nearly the exact attribution methodology at the annual level, we would not expect these small differences to impact our overall findings and conclusions. This study is limited using claims data to describe health status. Thus, the results regarding health status of the unattributed group should be interpreted as a likely underestimate, rather than necessarily reflecting better health. A similar limitation applies to our assessment of overall health care use among the unattributed group. However, we included these analyses to investigate if the unattributed group may use less ambulatory care but more emergency or inpatient care (eg, if a lack of outpatient care leads to higher-acuity utilization).

## Study results

Of 39M eligible beneficiaries, the vast majority (91%) could be attributed to a provider using the CPC+ rules. Most of these (68%) were attributed using the plurality of visits, meaning they had no CCM visit or AWV. Despite CCM coming first in the attribution hierarchy, only 2.4% (*n* = 852 825) were attributed this way compared to 29% attributed by AWV (*n* = 10 516 404). If the order were reversed, an additional 443 779 beneficiaries would be attributed by AWV.

**Figure 1.**
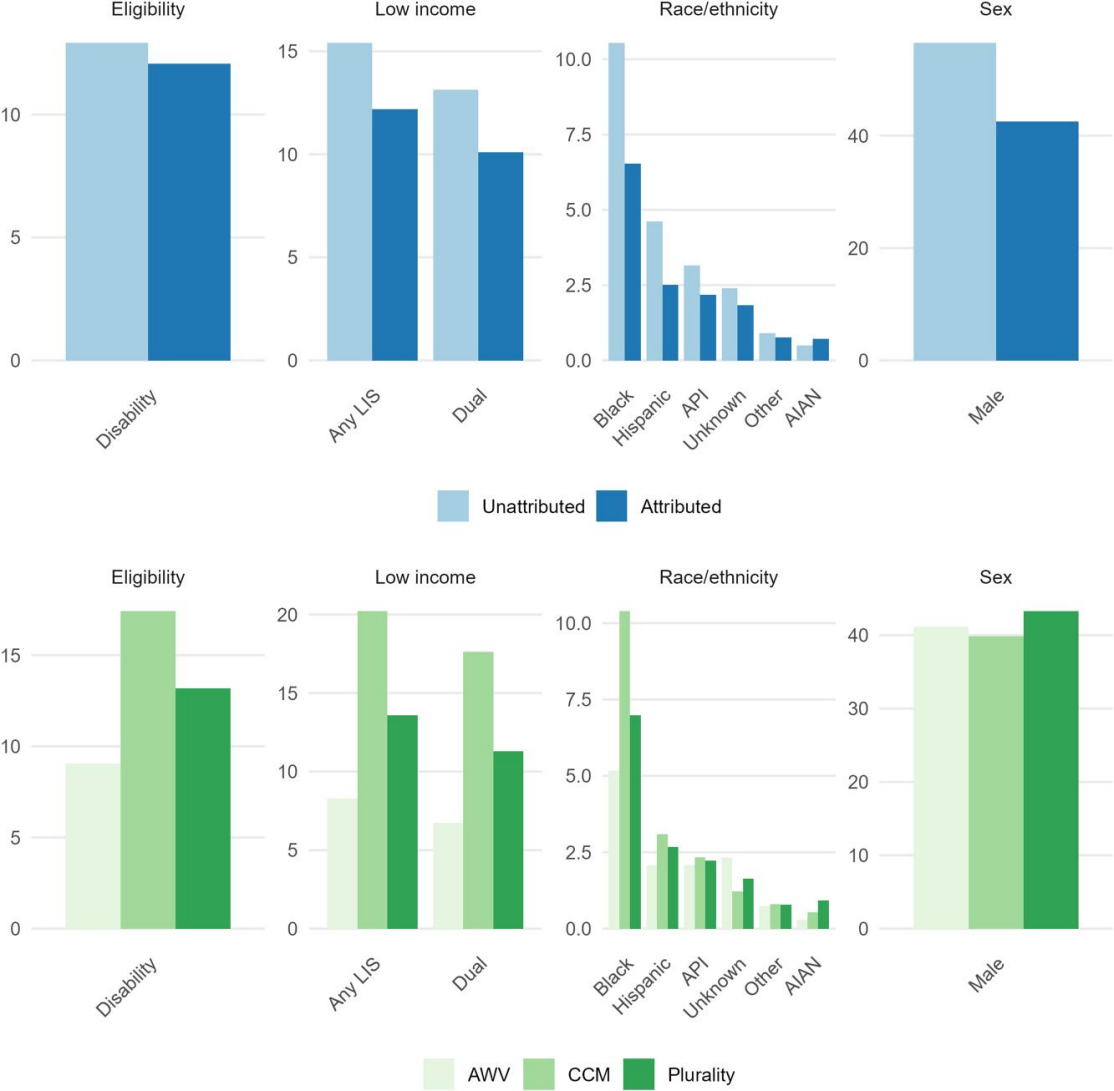
Demographics of beneficiaries by attribution status and method. Source: Authors*’* analyses of data from the Medicare Master Beneficiary Summary Base Enrollment File, Fee-For-Service Outpatient Claims Files, Fee-For-Service Carrier Claims Files, Master Beneficiary Summary File Chronic Conditions Segment, and Master Beneficiary Summary File and use segment, 2016-2021. Notes: Each bar shows the proportion of beneficiaries in the corresponding group with the labelled characteristic. The top panels show results for all beneficiaries eligible for attribution, separately by attribution status, while the bottom panels show results among attributed beneficiaries, separately by attribution method. Attribution using Annual Wellness Visits began in 2018; therefore, the characteristics of this group reflect the years 2018-2021. LIS: low-income subsidy; Dual: dual-eligible for Medicare and Medicaid; API: Asian or Pacific Islander; AIAN: American Indian or Alaskan Native; AWV: Annual Wellness Visit; CCM: Chronic Condition Management visit.

Unattributed beneficiaries (3.4M) were slightly younger (mean 72 vs 75 years) and more likely to be male (56% vs 43%), eligible for the low-income drug subsidy (15% vs 12%), dual eligible (13% vs 10%), and non-White (22% vs 15%), as shown in Figure 1. The starkest differences were in race and ethnicity. Unattributed beneficiaries comprised 10.5% Black and 4.6% Hispanic beneficiaries, compared to 6.5% Black and 2.5% Hispanic among those attributed. Compared to beneficiaries attributed by plurality of services, those attributed by AWV were less likely to be eligible due to disability (9% vs 13%), receive LIS (8% vs 14%), or be dual eligible (7% vs 11%), while those attributed by CCM had strikingly higher rates of these three indicators of social disadvantage (17% disability, 20% LIS, and 18% dual) and were more likely to be Black (10%) than those attributed by AWV (5%) or plurality (7%).

**Figure 2.**
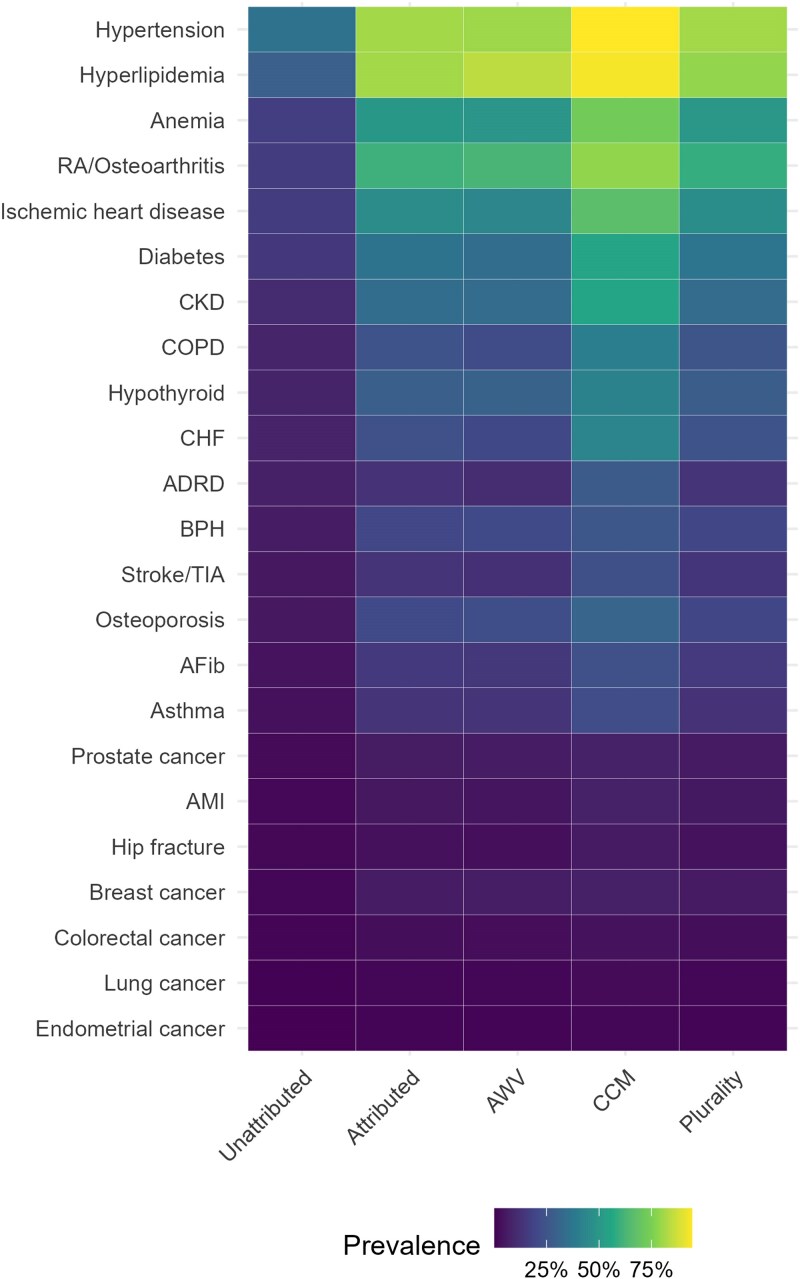
Chronic conditions of beneficiaries by attribution status and method. Source: Authors*’* analyses of data from the Medicare Master Beneficiary Summary Base Enrollment File, Fee-For-Service Outpatient Claims Files, Fee-For-Service Carrier Claims Files, Master Beneficiary Summary File Chronic Conditions Segment, and Master Beneficiary Summary File and use segment, 2016-2021. Notes: Each cell reflects the proportion of beneficiaries in the corresponding group with the row characteristic. The first three columns show results for all beneficiaries eligible for attribution, separately by attribution status, while the last three columns show results for attributed beneficiaries, separately by attribution method. RA: rheumatoid arthritis; CKD: chronic kidney disease; COPD: chronic obstructive pulmonary disease; CHF: congestive heart failure: ADRD: Alzheimer's disease and related dementias; BPH: benign prostatic hyperplasia, TIA: transient ischemic attack; AFib: atrial fibrillation; AMI: acute myocardial infarction; AWV: Annual Wellness Visit; CCM: Chronic Condition Management visit.

There were lower rates of chronic condition flags among the unattributed beneficiaries (Figure 2). The biggest differences were for the small number of beneficiaries attributed using CCM, who had higher rates of most chronic conditions, unsurprisingly.

**Figure 3.**
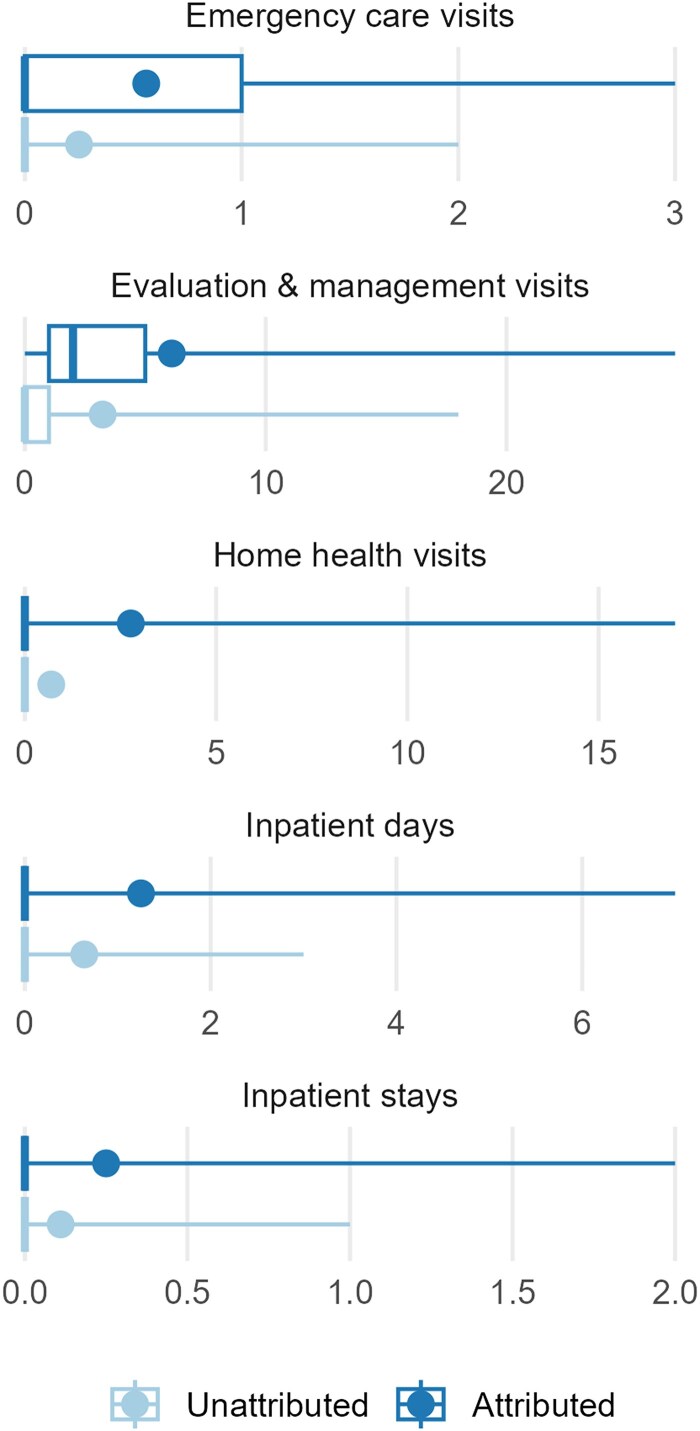
Health care use of beneficiaries by attribution status and method. SOURCE: Authors*’* analyses of data from the Medicare Master Beneficiary Summary Base Enrollment File, Fee-For-Service Outpatient Claims Files, Fee-For-Service Carrier Claims Files, Master Beneficiary Summary File Chronic Conditions Segment, and Master Beneficiary Summary File and use segment, 2016-2021. NOTES: Summary statistics of utilization for all beneficiaries eligible for attribution, separately by attribution status. Boxes extend from the 25th to 75th percentile, with a line at the median; whiskers extend from the 5th to 95th percentile; dots mark the means.

Consistent with having fewer chronic conditions, unattributed beneficiaries also used less health care, consistently across emergency, inpatient, home health, and E&M (Figure 3).

## Discussion

The attribution methodology used in payment and delivery models is critical for advancing health equity. When groups of individuals use less care (eg, due to access barriers), they are less likely to be attributed to models, thus reducing the representativeness of evaluations. In this study, we described the characteristics of attributed and unattributed beneficiaries using the CPC+ attribution methodology. While the CPC+ model ended in 2021, ongoing tests continue to use similar combinations of historical primary care claims combined with voluntary alignment.^17,18^

The majority of beneficiaries could be attributed to physicians using claims-based attribution while just under one in ten beneficiaries were unattributable. Compared to attributed beneficiaries, unattributed beneficiaries have more markers of exposure to social disadvantage (eg, low-income and minoritized race and ethnicity) but fewer chronic condition flags and visits. These latter differences are likely a combination of appropriate (having fewer health care needs) and inappropriate (facing barriers to accessing care) differences.

The fact that AWVs disproportionately contribute to the attribution of patients with fewer markers of social advantage may imply that this attribution method could lead to advantageous selection by providers. ACO leaders describe AWVs as a key strategy for their performance,^19^ and CPC+ providers specifically provided differentially more AWVs than matched comparison providers over the first 3 years of the model.^20^ Similar evidence from the Medicare Advantage program suggests that AWV may be used strategically by insurers.^21^ These visits may lead to attributed patients with greater social advantage and simultaneously provide opportunities to code diagnoses and thus increase capitated payments.

Our findings align with prior evidence that attribution using primary care claims may systematically leave out people with low income and from minoritized racial and ethnic groups.^10,22,23^ This is critical for model evaluations that may be unable to generate representative evidence that reflects the experiences of underserved beneficiaries.^2^ An important avenue for future research will be to evaluate how new attribution methodologies, particularly voluntary alignment and alternative service codes, and the hierarchy of attribution methodology, impact the inclusion and representation of historically underserved groups in payment and delivery models.

## Supplementary Material

qxag198_Supplementary_Data
